# Red Blood Cell Homeostasis: Mechanisms and Effects of Microvesicle Generation in Health and Disease

**DOI:** 10.3389/fphys.2018.00703

**Published:** 2018-06-08

**Authors:** Joames K. F. Leal, Merel J. W. Adjobo-Hermans, Giel J. C. G. M. Bosman

**Affiliations:** Department of Biochemistry, Radboud University Medical Center, Nijmegen, Netherlands

**Keywords:** red blood cell, microvesicles, membrane, aging, inflammation, autoimmunity, hemoglobin, oxidation

## Abstract

Red blood cells (RBCs) generate microvesicles to remove damaged cell constituents such as oxidized hemoglobin and damaged membrane constituents, and thereby prolong their lifespan. Damage to hemoglobin, in combination with altered phosphorylation of membrane proteins such as band 3, lead to a weakening of the binding between the lipid bilayer and the cytoskeleton, and thereby to membrane budding and microparticle shedding. Microvesicle generation is disturbed in patients with RBC-centered diseases, such as sickle cell disease, glucose 6-phosphate dehydrogenase deficiency, spherocytosis or malaria. A disturbance of the membrane-cytoskeleton interaction is likely to be the main underlying mechanism, as is supported by data obtained from RBCs stored in blood bank conditions. A detailed proteomic, lipidomic and immunogenic comparison of microvesicles derived from different sources is essential in the identification of the processes that trigger vesicle generation. The contribution of RBC-derived microvesicles to inflammation, thrombosis and autoimmune reactions emphasizes the need for a better understanding of the mechanisms and consequences of microvesicle generation.

## Introduction

Generation of microvesicles, i.e., extracellular vesicles that are shed from the plasma membrane (Raposo and Stoorvogel, 2013), constitutes an integral part of red blood cell (RBC) homeostasis, and is responsible for the loss of 20% of the hemoglobin and the cell membrane during physiological RBC aging *in vivo*, and the accompanying decrease in cell volume and increase in cell density (Willekens et al., 2003, 2008). The blood of healthy subjects contains approximately 1000 RBC-derived vesicles per microliter of plasma (Berckmans et al., 2001; Hron et al., 2007; Westerman et al., 2008; Willekens et al., 2008). The microvesicle hemoglobin composition suggests an enrichment of the irreversibly modified hemoglobins HbA1c and HbA1e2. Microvesicles contain various immunological recognition and removal signals (Willekens et al., 2008), that are responsible for a rapid elimination – probably within minutes – from the circulation (Willekens et al., 2005, 2008). Shedding of damaged cellular components by vesiculation prevents untimely removal of otherwise functional RBCs, as well as unwanted reactions of the hemostasis and immune systems. Thus, vesiculation couples general aging processes such as oxidation and glycation to organismal homeostasis. On the other hand, RBC-centered hemoglobinopathies such as sickle cell disease and thalassemia are accompanied by a substantial increase in microvesicle levels (Ferru et al., 2014; Camus et al., 2015). Also, infection of RBC with the malaria parasite *Plasmodium* induces vesicle formation. However, the underlying mechanism is likely to be strongly influenced by parasite-derived proteins, and therefore beyond the scope of this review (e.g., Mantel et al., 2013). Increased vesiculation is associated with systemic inflammation, which may be directly responsible for hemolysis and anemia (Dinkla et al., 2012b, 2016). Thus, microvesicles are part of the complex of interactions between RBCs and the organism (Bosman, 2016a,b). In this review, we aim to integrate the newest data on microvesicle composition and production in various conditions, in order to obtain more insight into the basic mechanisms underlying microvesicle generation, and the involvement of RBC-derived microvesicles in pathophysiology.

## Microvesicles *In Vivo*

**FIGURE 1:**
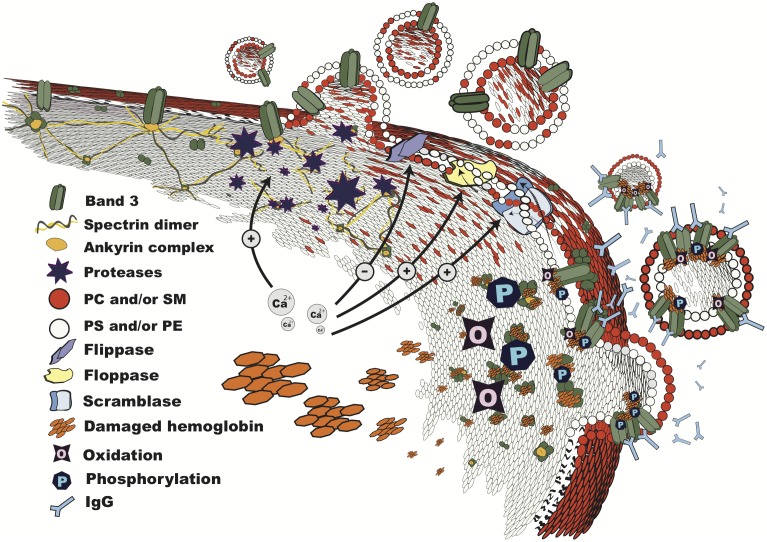
Vesiculation in progress. Structure of the RBC membrane during vesiculation, showing mechanisms involved in microvesicle shedding: breakdown of the cytoskeleton by calcium-dependent proteases; lipid bilayer rearrangement due to altered phospholipid transporter activities, which results in phosphatidylserine exposure; changes in band 3 configuration and distribution due to oxidation, binding of damaged hemoglobin, and phosphorylation, leading to loss of binding to the cytoskeleton at the ankyrin complex, recognition by IgG, and vesiculation. The order and interdependence of these processes are discussed in the text. PC, phosphatidylcholine; SM, sphingomyelin; PS, phosphatidylserine; PE, phosphatidylethanolamine.

Immunochemical analyses together with a proteomic inventory of RBC-derived microvesicles from the plasma of healthy individuals show the almost exclusive presence of the membrane proteins band 3 and actin (Bosman et al., 2012). Microvesicles are enriched in enzymes involved in redox homeostasis, i.e., glutathione S-transferase, thioredoxin and peroxiredoxin-1 and peroxiredoxin-2, and in ubiquitin. The hemoglobin composition of microvesicles isolated from plasma resembles that of the oldest cells, with an enrichment in irreversibly modified hemoglobins HbA1c and HbA1e2 (Willekens et al., 1997). Microvesicles display removal signals such as phosphatidylserine in the outer membrane layer and senescent cell-specific band 3 epitopes that are mainly found in the oldest RBCs (Willekens et al., 2008). Also, microvesicles contain the glycosylphosphatidylinositol (GPI)-anchored, complement-inhibiting proteins CD55 and CD59. These may not be in a functional configuration, as recently shown for the, similarly GPI-anchored enzyme acetylcholinesterase (Willekens et al., 2008; Freitas Leal et al., 2017). The striking absence of spectrin and ankyrin in microvesicles, together with immunoblot and proteomic patterns indicating extensive protein degradation in the oldest RBCs, as well as the aging-related increase in membrane-associated proteasome components (Bosman et al., 2012), all support a role for proteolytic breakdown of the band 3-ankyrin connection between the cytoskeleton and the lipid bilayer in the vesiculation process. However, the absence of especially spectrin in microvesicles isolated from the blood has led to alternative explanations for the aging-associated changes in RBC cell volume and density (Ciana et al., 2017a,b). The breakage of the band 3-ankyrin binding is predicted to cause a relaxation of the cytoskeletal spring and thereby spontaneous buckling of the lipid bilayer, resulting in evagination and vesiculation (Sens and Gov, 2007; Bosman et al., 2012) (**Figure 1**). Proteomic analysis of aging RBCs and RBC-derived microvesicles purified from the plasma support this model (Bosman et al., 2012), but do not provide many clues for the upstream processes, or for the relatively high concentration of actin in the microvesicles. The hemoglobin data, together with the accumulation of redox status-regulating enzymes, indicate that damage to hemoglobin may be such an upstream process, if not the primary trigger for vesiculation. A role for damaged hemoglobin in vesiculation is supported by the finding that the concentration of RBC-derived microvesicles is increased in the blood of patients with hemoglobinopathies (Westerman et al., 2008). The microvesicles from the blood of thalassemia patients contain high concentrations of oxidized, denatured alpha globin chains (Ferru et al., 2014). Also, these microvesicles contain enzymes involved in the maintenance of redox status such as catalase and peroxiredoxin-2, as well as large amounts of complement proteins and immunoglobulins (Ferru et al., 2014).

The protein composition of microvesicles from patients with membranopathies, i.e., abnormal RBCs such as elliptocytes and stomatocytes due to genetic aberrations in membrane proteins, is likely to differ from those of control RBCs. Actual data are lacking, but this prediction can be deduced from the effects of splenectomy on the membrane protein composition of spectrin/ankyrin-deficient and band 3-deficient spherocytes (Reliene et al., 2002; Alaarg et al., 2013). In RBCs from patients with thalassemia intermedia, hemoglobin damage may induce the formation of band 3 polymers, associated with increased phosphorylation that leads to a weakening of the band 3-ankyrin connection, resulting in microvesicle formation (Ferru et al., 2014). These observations, together with the vesiculation-reducing effect of p72Syk kinase inhibitors, not only support a central role for the binding of modified hemoglobin, possibly especially to oxidized and/or proteolytically degraded band 3 (Arashiki et al., 2013; Lange et al., 2014) in the vesiculation process (**Figure 1**), but also show the involvement of phosphorylation networks in RBC homeostasis. The involvement of various signaling pathways in RBC vesiculation was supported by the relative large numbers of signaling proteins in microvesicles obtained from the plasma of a healthy donor (Bosman et al., 2012), and in a pharmacological screening *in vitro* (Kostova et al., 2015).

## The Involvement of Microvesicles in Coagulation and Inflammation

Most RBC-derived microvesicles from healthy donors as well as from various patients expose phosphatidylserine, which promotes not only phagocytosis but also coagulation.

*In vitro*, thrombin generation through the intrinsic pathway has been shown to be induced by RBC-derived microvesicles derived from sickle cell patients, storage units, or after treatment with a calcium ionophore (van Beers et al., 2009; van der Meijden et al., 2012; Rubin et al., 2013). In addition, correlations have been reported between the number of phosphatidylserine-exposing, RBC-derived microvesicles, and thrombin generation in sickle cell patients (van Beers et al., 2009; Gerotziafas et al., 2012). Microvesicles may also disturb anticoagulation reactions of the protein C system, possibly through binding of protein S (e.g., Koshiar et al., 2014). Increases in RBC-derived microvesicles in sickle cell disease and thalassemia patients is often accompanied by a decrease in deformability and hemolysis, which may as such constitute a risk factor for thrombosis.

Phagocytosis-triggered monocyte activation may induce proinflammatory and procoagulant endothelial cell responses (Straat et al., 2016). Thrombin may promote inflammation by activation of the complement system, e.g., by acting as C3 or C5 convertase (Zecher et al., 2014). RBCs of patients with paroxysmal nocturnal hemoglobinuria (PNH) lack the GPI-anchored proteins CD55 and CD59 that protect against complement activation-associated hemolysis. GPI-anchored proteins may be involved in raft formation (Salzer and Prohaska, 2001), and their absence may be directly responsible for the release of relatively high numbers of RBC-derived microvesicles with procoagulant activity in PNH patients *in vitro* (Hugel et al., 1999; Kozuma et al., 2011; Devalet et al., 2014). In addition, microvesicles scavenge NO almost as fast as free hemoglobin and much faster than RBCs, which may impair vasodilation (Donadee et al., 2011). This effect is already detectable with the number of microvesicles present in one transfusion unit (Liu et al., 2013).

## The Role of the Spleen

The spleen facilitates vesiculation, as apparent from the retention of microvesicles in RBCs in asplenic individuals. In these individuals, the normal aging-related decrease in total RBC hemoglobin is absent, due to an increase in HbA1c (Willekens et al., 2003). In patients with spherocytosis, splenectomy increased RBC deformability *in vitro*, probably by inhibiting spleen-mediated microvesicle shedding (Reliene et al., 2002). The molecular mechanism underlying vesiculation in the spleen is unknown, but may involve a combination of biochemical and biophysical stress. Recent model simulations support the involvement of degraded hemoglobin in reducing the cytoskeleton/membrane connection, thereby promoting microvesicle shedding during splenic flow (Zhu et al., 2017). Thus, the mechanical and biochemical circumstances in the spleen, together with the presence of specialized macrophages, may make the spleen a microvesicle-based quality control and repair system. This emphasizes the importance of establishing the functionality of the spleen, especially in the study of diabetic control (Willekens et al., 2003). Also, the notable paucity of data for microvesicles generated *in vivo* warrants a more detailed investigation on the fundamental and clinical relationship between splenectomy or functional asplenia, RBC-derived microvesicles and RBC homeostasis (Wernick et al., 2017).

## Microvesicles *In Vitro*

Vesiculation also occurs during storage of RBCs in the blood bank. Storage microvesicles contain removal signals such as phosphatidylserine in the outer layer of their membrane and degraded as well as aggregated band 3 molecules, similar to microvesicles in the circulation (Bosman et al., 2008; Willekens et al., 2008). In blood bank microvesicles, the number of carbonyl groups is increased relative to RBC membranes, possibly due to the accumulation of oxidized membrane proteins band 3, actin and protein 4.1 (Bosman et al., 2008; Kriebardis et al., 2008; Delobel et al., 2016). This is accompanied by a correlation between oxidized cell constituents and vesiculation during storage (D’Alessandro et al., 2017). Blood bank microvesicles are immunologically active, as they contain immunoglobulins and complement factors, derived from the plasma fraction of the storage fluid (Bosman et al., 2008; Kriebardis et al., 2008). Also, storage microvesicles are readily recognized by pathological autoantibodies from patients with autoimmune hemolytic anemia (Dinkla et al., 2012a). These data indicate that the coupling of removal of damaged components from the RBC to their removal from the circulation is a general phenomenon for RBC-derived microvesicles. The enrichment of the GPI-anchored proteins acetylcholinesterase and CD55, as well as raft-associated forms of stomatin and the flotillins in storage microvesicles, indicates that lipid-related changes in membrane organization are involved in vesiculation during storage (Bosman et al., 2008; Salzer et al., 2008). The underlying mechanism has been proposed to be revolving around membrane budding and fission. This could be triggered by the loss of binding between cytoskeletal and membrane proteins, followed by large-scale separation of various lipid phases that may be formed by membrane protein-stabilized microdomains (Salzer et al., 2008). The loss of interaction between the cytoskeleton and cell membrane may be triggered by oxidized hemoglobin, similar to what may happen *in vivo*. Indeed, accumulation of oxidized hemoglobin residues during storage is accompanied by their enrichment in microvesicles (Wither et al., 2016). This role of hemoglobin in microvesicle formation is supported by the observation that, in the early phase of storage, a significant amount of hemoglobin is associated with the lipid bilayer in microvesicles (Szigyártó et al., 2018). There is a shortage of detailed quantitative and qualitative information on the primary triggers driving microvesicle production *in vitro*. The available data, albeit mostly showing associations, support a role of phosphorylation and rearrangement of band 3. For example, inhibition of tyrosine dephosphorylation not only induces RBC shapes such as echinocytes, which indicates a loss of interaction between the cytoskeleton and the lipid bilayer but also stimulates microvesicle production *in vitro* (Ferru et al., 2011, 2014; Cluitmans et al., 2016). In the misshapen cells found in patients with neuroacanthocytosis, disturbed phosphorylation and altered cell morphology are accompanied by disturbed microvesicle generation (Bosman and De Franceschi, 2008). Phosphorylation of band 3 is associated with clustering and correlates with microvesicle formation during storage and in the RBCs of patients with thalassemia intermedia (Ferru et al., 2014; Azouzi et al., 2018). Similar effects are observed upon treatment of RBCs with agents that induce aggregation of band 3 (Ferru et al., 2011; Cluitmans et al., 2016). A well-known stimulus for microvesicle formation *in vitro* is an artificial increase in intracellular calcium concentration. However, the protein composition of calcium-induced microvesicles differs from storage or blood microvesicles, e.g., the content of membrane proteins, the presence of band 3 aggregates and breakdown products, and of raft-associated proteins (Bosman et al., 2008; Salzer et al., 2008; Prudent et al., 2015). This indicates that alterations in intracellular calcium concentrations are not primary factors in microvesicle generation *in vivo*, nor in the blood bank.

## Mechanisms of Vesiculation: Involvement of Lipids

The few data that are available indicate that disturbances of the organization of the lipid part of the cell membrane may play a role in the vesiculation process. The RBC membrane contains sphingomyelin/cholesterol-enriched as well as cholesterol-enriched domains that are associated with high-curvature areas. Since these domains become associated with budding membrane areas during storage at 4°C, they have been speculated to be specific sites of microvesicle generation (Leonard et al., 2017). However, RBCs and microvesicles obtained during storage in blood bank conditions showed no significant differences in the main phospholipid classes (Laurén et al., 2018). This included the lack of enrichment of the raft-associated lipids cholesterol and sphingomyelin. Thus, lipid-involving reorganizations in the RBC membrane may be instrumental in microvesicle generation, but they do not seem to result in significant alterations in microvesicle lipid composition. Changes in membrane lipid organization, such as an increase in exposure of phosphatidylserine and/or phosphatidylethanolamine, may promote vesiculation during storage (Verhoeven et al., 2006; Larson et al., 2017). However, severe disruptions of the protein-protein interactions, that are associated with altered RBC morphology, may induce increased microvesicle generation, but are not always accompanied by increased phosphatidylserine exposure (Cluitmans et al., 2016) (**Figure 1**). In this context, it should be emphasized that not all RBC-derived microvesicles expose detectable amounts of phosphatidylserine (Willekens et al., 2008; Nielsen et al., 2014). Disruption of the lipid bilayer, e.g., by treating RBCs with sphingomyelinase, strongly catalysed microvesicle generation *in vitro*. This process was accompanied by the appearance of CD59 and stomatin clusters in the RBCs, supporting a role for lipid rearrangement in microvesicle formation. The sphingomyelinase-induced microvesicles were much more heterogeneous in phosphatidylserine exposure and glycophorin A content than the microvesicles generated by spontaneous vesiculation, indicating the involvement of different mechanisms (Dinkla et al., 2012b). Thus, changes in lipid organization may facilitate microvesicle formation, but may not constitute the primary mechanism in most physiological conditions.

## Mechanisms of Vesiculation: Comparison With Exosome Formation

All reports on RBC-derived microvesicle composition, especially in combination with the aging-associated changes in the RBC membrane proteome, indicate the involvement of proteins that are involved in the release of exosomes as well (Raposo and Stoorvogel, 2013). This includes small GTPases, lipid raft-associated proteins such as acetylcholinesterase and flotillins, and annexins (Bosman et al., 2012; Raposo and Stoorvogel, 2013; Prudent et al., 2015). Although it is not clear how cytosolic components end up in exosomes, the mechanisms by which cytosolic molecules are recruited into RBC-derived microvesicles may be similar to those involved in exosome generation, as indicated by the presence of various chaperone proteins (Bosman et al., 2012; Raposo and Stoorvogel, 2013). The molecular details of the mechanisms underlying microvesicle generation in other cell types are largely unknown. Incorporation of the available data on RBC-derived microvesicles into the catalog ‘Vesiclepedia’ (Kalra et al., 2012) may be a worthwhile first step toward further elucidation of the mechanism of microvesicle in RBCs, as well as in other cell types. A comparison of RBC microvesicle data with the already available data on RBC exosomes that are shed by reticulocytes from human cord blood (Chu et al., 2018) will facilitate the identification of the molecular mechanisms involved in various types of vesiculation *in vivo*. Already, endocytosis and autophagy have been involved in the disappearance of CD71 and other membrane proteins during reticulocyte maturation *in vitro* (Griffiths et al., 2012). Such an approach profits from the possibility that RBCs create to study exosome as well as microvesicle formation during differentiation and aging in the same cell, that has a relatively homogeneous and well-charted membrane system.

## From Mechanism to Marker to Medicine

Red blood cells form microvesicles in response to a variety of physiological and pathological triggers. Although the inventory of the composition of microvesicles generated in different circumstances is far from complete, the available data indicate that they all are enriched in damaged RBC components, depending on the various stimuli (**Figure 1**). This suggests that an exhaustive study of RBC-derived microvesicles will offer insights into the molecular mechanisms of their generation *in vivo*, and thereby into the physiological and pathological triggers. Also, RBC-derived microvesicles may constitute a model for the study of the biological, biophysical and clinical properties of microvesicles in general. This model will benefit from the comparison of the composition and characteristics of RBC-derived microvesicles with microvesicles generated by other cell types, and with exosomes. RBC-derived microvesicles are potentially sensitive and specific biomarkers for the clinical severity of RBC-centered diseases such as sickle cell disease, thalassemia or spherocytosis (Reliene et al., 2002; Hebbel and Key, 2016; Kittivorapart et al., 2018). Also, microvesicles may reveal the activity as well as the clinical consequences, such as anemia or thrombosis, of systemic processes, such as inflammation. In addition, RBC-derived microvesicles may be useful in the transfer of surface proteins, as has been shown in the ‘painting’ of RBCs of PNH patients with the complement-protecting proteins CD55 and CD59 (Sloand et al., 2004).

Where RBC-derived microvesicles may be actively involved in pathology, e.g., by their procoagulant, proinflammatory or autoimmune activity (Sadallah et al., 2008), pharmacological prevention of the formation of harmful microvesicles may become of clinical importance. The recent finding that inhibition of sphingomyelinase attenuated lung inflammation caused by infusion of stored RBC-derived microvesicles (Hoehn et al., 2017), supports this notion. Thus, on one hand, microvesicle shedding may prevent the untimely removal of functional RBCs in physiological conditions. On the other hand, in pathological conditions, prevention of vesiculation following splenectomy may have beneficial effects, or prevent a pathological immune reaction, for example after massive or frequent RBC transfusion in compromised, transfusion-dependent patients.

## Author Contributions

GB conceived the topic and wrote the final version. GB, JL, and MA-H wrote parts of the manuscript.

## Conflict of Interest Statement

The authors declare that the research was conducted in the absence of any commercial or financial relationships that could be construed as a potential conflict of interest.
